# Emergency committee recommendations on mpox – what’s next?

**DOI:** 10.2471/BLT.23.290134

**Published:** 2023-05-01

**Authors:** Rosamund F Lewis, Krutika Kuppalli, Ana Hoxha, Meg C Doherty, on behalf of WHO mpox response incident management team

**Affiliations:** a Epidemic and Pandemic Prevention and Preparedness Department, Health Emergencies Programme, World Health Organization, Avenue Appia 20, 1211 Geneva 27, Switzerland.; b Alert and Response Coordination Department, World Health Organization, Geneva, Switzerland.; c Department of Global HIV, Hepatitis and Sexually Transmitted Infections Programmes, World Health Organization, Geneva, Switzerland.

In May 2022, an unprecedented global outbreak of mpox emerged,^1^ prompting the Director-General of the World Health Organization (WHO) to declare a Public Health Emergency of International Concern.^2^ By 18 April 2023, 110 countries reported 87 039 cases of mpox and 120 related deaths to WHO, with 28 countries still reporting cases and sustained transmission, most of them outside of the known ecological niche of the virus.^3^ In February 2023, WHO convened the fourth meeting of the International Health Regulations (2005) (IHR) Emergency Committee for mpox.^4^ Members of the committee commended progress in containing the outbreak and noted ongoing concerns: many countries continued to report cases, primarily among men who have sex with men; in some countries mpox appeared among vulnerable populations with limited access to testing, vaccines and therapeutics; uncertainty that behaviour modification would last; declining surveillance and case reporting to WHO along with surveillance challenges in low-income settings; and limited evidence regarding effectiveness of vaccines and therapeutics, or the duration of vaccination-induced or post-infection immunity.^4^

Therefore, the Director-General of WHO concurred that the outbreak remained a Public Health Emergency of International Concern. WHO advised Member States to strengthen key areas of readiness and response to stop the outbreak, and to strive towards mpox elimination or control based on specific epidemiological contexts.^5^ To achieve these goals, the committee recommended five key areas of action.

First, as countries monitor the outbreak and develop elimination and control plans, surveillance must be sustained and strengthened. As the number of reported mpox cases continues to decline globally, all Member States should sustain national surveillance for suspected cases and continue to report confirmed and probable cases and deaths.^3^ Doing so will allow for rapid detection and investigation of new cases and clusters of mpox. Strengthening surveillance also remains critical to understanding and controlling mpox in affected countries in the African region.

Second, integrate mpox surveillance, detection, prevention and care into primary care and sexual health services. Among mpox cases with known human immunodeficiency virus (HIV) status, approximately half are living with HIV, and advanced infection is an important risk factor for severe complications and death related to mpox.^3^^,^^6^ Strong HIV prevention and care is a central pillar of mpox preparedness and response as both infections can spread through sexual networks. Person-centred delivery of care for mpox in sexual health and HIV programmes can help eliminate stigma and discrimination, support access to services and improve outcomes. Engagement and consultation with affected communities is critical to ensuring that risk communications and health services are timely, effective, appropriate and acceptable, and reach people.

Third, enhance global commitment and cooperation to facilitate equitable access to diagnostics, vaccines and therapeutics. The mpox outbreak has again highlighted global inequities as vulnerable populations, ethnic minorities and persons living in low-income countries continue to face barriers to care.^7^ Working with partners to implement harmonized strategies among regions, coordinating and scaling up allocation mechanisms, establishing target product profiles to stimulate research and development, technology transfer and other measures are essential to advance health equity and global health security.

Fourth, strengthen and support capacity in resource-constrained settings, including for One Health approaches and for research at the animal-human interface, where zoonotic spillover is known or suspected to occur. Understanding of animal reservoirs and transmission to humans remains limited. Identifying factors that contribute to amplification of outbreaks or extended chains of transmission is also critical. Improving multisectoral collaboration to address socioeconomic and behavioural risk factors will support research to characterize animal-to-human and human-to-human transmission in different contexts.

Fifth, implement a strategic and coordinated research agenda to ensure ongoing evidence generation. Strategic areas of focus include: investigating the origins of monkeypox virus; characterizing epidemiology and dynamics of viral transmission including for the global outbreak of clade IIb.B.1, which has never been found in animals;^8^ understanding the clinical characteristics of the disease;^9^ describing social determinants for affected groups including the impact of behaviour change;^10^ and investing in the development of countermeasures such as rapid diagnostics and next generation therapeutics and vaccines.^11^

To achieve these goals, WHO will propose a global strategy for mpox elimination and control and approaches to support countries in all regions to develop their own integrated plans. This dual approach will help countries set clear targets for stopping human-to-human transmission; maintain high-quality indicator-based surveillance and event-based monitoring; support broader efforts towards epidemic and pandemic preparedness; and ensure adaptation of public health and communication strategies for specific contexts and groups at risk, particularly where mixed modes of transmission are observed.

We have the tools to end this outbreak and ensure elimination of human-to-human transmission and ongoing control of mpox. To do this, the global health community must come together and invest in the key areas highlighted.
